# Comparative Efficiency Analysis of OECD Health Systems: FDH vs. Machine Learning Approaches with Efficiency Analysis Trees (EAT and RFEAT)

**DOI:** 10.1186/s12962-025-00607-x

**Published:** 2025-02-22

**Authors:** Yejin Joo

**Affiliations:** Department of Economics, Seoul National University, 599 Gwanak-ro, Gwanak-gu, Seoul, 151-742 Republic of Korea

**Keywords:** OECD, Efficiency Analysis Trees, Random Forest for Efficiency Analysis Trees, Inefficiency underestimation, Health system

## Abstract

**Background:**

As health expenditure continues to rise due to income growth, technological advancements, and an aging population, it has become increasingly important to accurately measure and improve the efficiency of health systems. This is because financial resources are limited, and the allocation of resources can significantly influence the quality of health systems and health outcomes.

**Methods:**

This study applies machine learning techniques—Efficiency Analysis Trees (EAT) and Random Forest for Efficiency Analysis Trees (RFEAT)—to evaluate the efficiency of health systems in 36 OECD countries, comparing the results with those from the traditional free disposal hull (FDH) method.

**Results:**

Analysis shows high discrimination power in the order of RFEAT, EAT, and FDH. The correlation in efficiency rankings shows more than 80% similarity between RFEAT and EAT, while both show less than 80% similarity with FDH. According to RFEAT estimates, the countries with the highest efficiency are South Korea, Switzerland, and Costa Rica, whereas the United States, Lithuania, and Latvia are identified as the least efficient. The group-level analysis reveals that Asian countries, on average, perform more efficiently followed by Oceania, Europe, and the Americas. The groups with higher out-of-pocket healthcare expenditures per capita tend to show slightly better efficiency and the group with the smallest elderly population proportion exhibits the highest average health system efficiency.

**Conclusion:**

Traditional methods like FDH are prone to inefficiency underestimation, especially in small samples with multiple variables. This study demonstrates the potential of machine learning approaches like EAT and RFEAT to provide more reliable efficiency estimates. These methods can help policymakers make better resource allocation decisions by mitigating inefficiency underestimation and offering greater discrimination power.

## Introduction

From 2000 to 2019, the ratio of health expenditures to Gross Domestic Product (GDP) in Organization for Economic Cooperation and Development (OECD) countries steadily increased from approximately 7% to 9%, driven by factors such as rising incomes, technological advances, and an aging population [1]. The COVID-19 pandemic further accelerated this trend, contributing to an additional increase compared to pre-pandemic levels [1]. Absent significant policy changes, the OECD forecasts that this growth will persist, with health spending projected to reach 11.2% of GDP by the year 2040 [1]. However, fiscal constraints have emerged as a critical barrier to expanding health expenditure, leading to a growing focus on improving the quality of health systems to ensure sustainable healthcare financing.

The quality in health system is assessed through metrics in eight domains: effectiveness (evaluating prevention, treatment, and outcomes for various diseases or demographic groups), patient safety, timeliness, patient-centeredness, coordination of care, efficiency, infrastructure of health and medical system, and accessibility of health and medical services [2]. Efficiency, one of measures used to estimate the quality of health and medical system, reflect health-improving outcomes relative to costs. Poor performance in the health system is often attributed to inefficiencies, and inefficient health systems necessitate higher health budget expenditures to achieve the same health outcomes [3]. Therefore, for sustainable public finance and to address increasing healthcare demands, enhancing efficiency is necessary [4]. To improve efficiency by properly allocating limited resources, accurate measurement and analysis of efficiency are crucial.

The methods employed in previous studies to estimate efficiency by constructing a frontier can be broadly categorized as parametric and nonparametric approaches. The nonparametric model (deterministic frontier model) assumes that all observations in the data sample are included in the production set, whereas the parametric model (stochastic frontier model) allows for the possibility that observations in the data sample may lie outside the production set due to random noise. A deterministic approach has been criticized for not permitting stochastic error terms, sensitivity to sampling noise in small sample sizes, and vulnerability to outliers [5, 6]. However, it has the advantage of allowing for the specification of multiple output variables. Additionally, it is free from the arbitrariness associated with the model form and the distribution of the inefficiency term, which are drawbacks of the stochastic frontier model [7, 8]. 

Free Disposal Hull (FDH) and Data Envelopment Analysis (DEA) are the predominant nonparametric frontier estimation methodologies extensively employed in prior academic research. FDH, introduced by Deprins and Simar [9], is based on free disposability, a deterministic approach, and minimal extrapolation, constructing stepwise frontiers solely from observed data points. DEA, developed earlier by Charnes, Cooper, and Rhodes [10], shares these principles but differs by incorporating convexity and linearity assumptions, resulting in a piecewise linear frontier. When sample sizes are insufficient relative to dimensionality,^1^ both methods may produce efficiency frontiers that lie below the theoretical boundary, leading to an underestimation of technical inefficiency (Overfitting in DEA/FDH).^2^

While undervaluation of inefficiency may not always be deemed detrimental, it can lead to the following problems: First, efficiency overestimation tends to occur significantly in small datasets or high-dimensional data, which undermines the accuracy of performance evaluation results. If such biased evaluations are reflected in policymaking, they risk distorting policy implications and leading to suboptimal resource allocation decisions. This issue is particularly critical when biased efficiency scores are used in secondary analyses, as it may introduce further distortions into subsequent results. Second, DEA and FDH models, often classify a large number of Decision Making Units (DMUs) with the same efficiency score. This reduces their ability to clearly distinguish between efficient and inefficient DMUs, thereby limiting their practical applicability for policy implementation. To address these critical problems, various methods have been developed to mitigate the issue of upward bias in efficiency estimates [13]. These advancements have primarily focused on integrating advanced techniques into traditional models, highlighting the need for further exploration of innovative approaches (see Literature Review).

Building on this need, this study is the first to apply two machine learning techniques proposed by Esteve et al. [11, 12], Efficiency Analysis Trees (EAT) and Random Forest for Efficiency Analysis Trees (RFEAT), in the health sector.^3^ The two machine learning techniques have been proven to outperform FDH in mitigating the inefficiency underestimation through Monte Carlo simulations [11, 12]. It examines how the efficiency of health systems in 36 OECD countries varies when estimated using the traditional FDH and two machine learning approaches, and further analyzes the results obtained through the RFEAT.

Although machine learning techniques are not yet widely applied in estimating the efficiency of national health systems, efficiency estimation methodologies have continued to evolve, incorporating these approaches to address key limitations [13]. Machine learning models, such as DEA combined with Support Vector Machines (SVM) or Artificial Neural Networks (ANN), have emerged as promising tools for capturing the nonlinear relationships between input and output variables [14]. These approaches represent significant progress in overcoming the limitations of traditional methods, but challenges remain, particularly in handling small datasets, mitigating the inefficiency underestimation, and ensuring interpretability.

To address these challenges, this study selected and applied EAT and RFEAT, to estimate the efficiency of health systems in 36 OECD countries. Specifically, these methodologies were chosen for the following reasons. First, random forest often demonstrates superior predictive performance compared to SVM and ANN in small- to medium-sized datasets. SVM performance can degrade due to overfitting in small datasets, while ANN typically require large datasets to achieve optimal performance [15]. Second, RFEAT mitigates overfitting and reduces upward bias in efficiency estimates through ensemble methods such as bagging and random subspace techniques. Similarly, EAT employs cost-complexity pruning to address overfitting and its associated upward bias [16]. Correcting upward bias in efficiency estimates also streamlines the recalibration process when new data affecting the frontier is introduced, saving significant time and effort. In contrast, methods combining SVM or ANN with FDH/DEA do not structurally address this issue.

Third, random forest is robust to noise and outliers, a critical advantage in real-world datasets. This robustness stems from its ensemble nature and randomized feature selection, which enhance stability and reliability [15]. In comparison, SVM and ANN are more sensitive to noisy data, often requiring additional preprocessing steps such as normalization or outlier removal to maintain performance [17]. Finally, RFEAT and EAT provide variable importance metrics, offering insights into the factors influencing the efficiency frontier. EAT further enhances interpretability by using decision trees for model results. In contrast, SVM and ANN are often considered “black box” models, making their outcomes more challenging to interpret^4^ [18]. Additionally, random forest benefits from the general advantages of ensemble learning, including stable performance, robustness to irrelevant predictors, and reduced model tuning requirements [15].

The structure of the paper is as follows: Section “Literature review” reviews the literature on national health system efficiency estimation and examines machine learning-integrated methodologies currently utilized in other disciplines, with potential applicability to health system efficiency analysis. Section “Methodology” discusses methodologies such as FDH, Classification And Regression Tree (CART), EAT, and RFEAT for estimating production frontiers and outlines the dataset. Section “Application in OECD Countries” presents the empirical analysis results, covering the ranking of input variable importance, sensitivity analysis, hyperparameter optimization strategies, and the convergence patterns of Out-of-Bag (OOB) errors in RFEAT. Section “Discussion” offers a critical discussion of the study's findings, while Section “Conclusion” synthesizes the key conclusions and proposes avenues for future research.

### Literature review

In the health sector, nonparametric methods such as FDH and DEA have been applied to estimate the efficiency of national health systems. These studies primarily used output variables such as life expectancy or infant survival rate. A study by Önen and Sayin estimated the efficiency of health systems in 34 OECD countries using DEA, with life expectancy and infant survival rate as outputs, and hospital beds, medical staff, and nurses per 1000 people as inputs. They found that the classification of efficient and inefficient countries did not align with the categorization of developed and developing countries [19]. Another DEA analysis by Ahmed et al. compared the health systems of Asian countries, using life expectancy at birth and infant mortality as outputs, and per capita medical spending as an input. Censored Tobit and smoothed bootstrap approach were applied for the second stage analysis. The analysis revealed that out of 46 countries, 42 were inefficient in terms of resource use in their health systems, with most of the efficient countries were in the high-income group, and only one in the lower-middle-income group [20].

An analysis of the efficiency of 17 EU member states conducted by Dincă, G. et al. using DEA methods identified Sweden, the UK, and Romania as having the most efficient health systems [21]. In a comparison of 140 countries using output-oriented DEA method, Zarulli et al. estimated that, on average, an improvement of 5.47 years in life expectancy was possible. The least efficient countries could improve up to 11.78 years, while the most efficient countries could gain approximately 0.83 years [22].

However, the traditional efficiency estimation models used in the aforementioned studies to estimate health system efficiency suffer from the issue of upward bias in efficiency estimates when the number of observations is small relative to the number of predictors. To address this problem, biased-corrected bootstrapping techniques have been developed [23]. Bootstrapping techniques also facilitated the estimation of confidence intervals [23]. This bias-corrected bootstrapping method has subsequently been employed in numerous studies estimating health system efficiency [24–28].

Kim and Kang used the Simar and Wilson bootstrapped DEA analysis to estimate the health system efficiency of 170 countries which were divided into four groups [26]. This study used life expectancy and under-five mortality rate as outputs, and public health expenditure and average years of schooling for women aged 15 or older as inputs. The results showed that most countries were inefficient in maximizing the use of input resources for the given level of output. While high-income countries had relatively higher average efficiency, only 16.7% of countries were found to be efficient, with Asian countries being relatively more efficient [26].

A study from Pérez-Cárceles et al. used bias-corrected DEA to analyze the health systems of Europe and Central Asia using life expectancy and infant survival rate as outputs and medical expenses, healthcare professionals, and hospital beds as inputs [27]. The results showed that when interpreted with bias-corrected efficiency, the rankings of healthcare systems changed, and the factors affecting the efficiency also differed. Additionally, the study found that lifestyle factors, policy organization, and geographic location influenced efficiency outcomes [27].

Garcia-Escribano et al. used bootstrap-DEA proposed by Simar and Wilson in 2007 to derive biased-corrected efficiency scores [25]. In this study, life expectancy at birth for the latest year available was used as an output and the averaged total health expenditures per capita over five-year period was employed as an input. Health-adjusted life expectancy (HALE) was used for an output variable of robustness check. They showed addressing overall income inequality and controlling corruption had a positive impact on reducing inefficiency in national health systems [25].

Although not yet widely applied in national health system efficiency research, new machine learning-based efficiency estimation methods have been developed to leverage the strengths of machine learning, while some of them address inefficiency underestimation. These methods have been applied in various other fields [13]. For instance, non-parametric methods combined with machine learning techniques, such as CART [29–31], ANN [14, 32], and SVM [14] have been developed and applied in analyses. Furthermore, efficiency determinants in industrial-scale co-digestion facilities in the U.S. and Germany have been evaluated using DEA combined with stochastic gradient boosting. Farm performance efficiency has been assessed using a DEA-Random Forest methodology, while the performance of Tunisian secondary schools has been analyzed using a hybrid model combining DEA, regression trees, and Random Forest [33–35].

## Methodology

### Free disposal hull (FDH) and efficiency analysis trees(EAT)

Free Disposal Hull (FDH), introduced by Deprins and Simar [9], is a nonparametric efficiency estimation method based on three microeconomic premises: free disposability, a deterministic model, and the principle of minimal extrapolation. While minimal extrapolation ensures conservative efficiency estimation, it often leads to inefficiency underestimation. In contrast, EAT overcomes this limitation by utilizing a machine learning framework that eliminates reliance on minimal extrapolation, thereby reducing inefficiency underestimation.

EAT, based on the CART framework, extends the capabilities of traditional decision trees to construct efficiency and production frontiers [11]. CART, proposed by Breiman et al. [36], functions as a nonparametric classification model for categorical response variables and as a regression model for numerical ones. While it does not assume disposability, CART’s stepwise output is conceptually similar to the stepwise efficiency frontier of FDH. It evaluates input variables and thresholds at each split, selecting those that minimize the sum of mean squared errors (MSE) of the child nodes to construct regression trees. This process repeats recursively until no significant splits remain or a stopping criterion is met. However, CART-generated trees often overfit due to their size and overly optimistic estimates. Breiman et al. [36] addressed this issue through cost-complexity pruning based on cross-validation.

Although CART is not inherently designed for efficiency analysis due to its lack of disposability assumptions, EAT adapts its framework to incorporate disposability and determinism for constructing efficiency frontiers. Additionally, unlike traditional nonparametric methods, which provide limited insights into the significance of input variables, EAT identifies their relative importance in predicting outputs.

Despite its advantages, EAT has limitations, such as sensitivity to noise and irrelevant input variables, which can lead to instability and reduced predictive accuracy [37]. Furthermore, its performance declines in scenarios with complex input variable interactions [38]. To address these challenges, RFEAT extends the Random Forest method [16], tailoring it for production frontier analysis.

Each n DMUs, $DM{U}_{i}=\left({{\varvec{x}}}_{i},{{\varvec{y}}}_{i}\right), i=1,\cdots ,n$, consumes inputs ${{\varvec{x}}}_{i}=\left({x}_{i1},\cdots {x}_{im}\right)\in {R}_{+}^{m}$ to produce outputs ${{\varvec{y}}}_{i}=\left({y}_{i1},\cdots {y}_{is}\right)\in {R}_{+}^{s}$. A dataset $\aleph$ comprising entities $\left({{\varvec{x}}}_{1},{{\varvec{y}}}_{1}\right),\dots ,\left({{\varvec{x}}}_{n},{{\varvec{y}}}_{n}\right)$ is given, where ${{\varvec{x}}}_{i}\in {R}_{+}^{m},{{\varvec{y}}}_{i}\in {R}_{+}^{s}, i=1,\dots ,n$.

The error for node t is calculated as the proportion of total observations in $t$, weighted by the MSE at the node:

1 $$R\left(t\right)=\frac{n\left(t\right)}{N}\cdot MSE\left(t\right)=\frac{1}{N}\cdot \sum_{\left({x}_{i},{y}_{i}\right)\in t}{{(y}_{i}-\widehat{y}(t))}^{2}$$

Here, the MSE reflects the squared difference between the observed values and the predictions at node $t$. The error of the entire tree $T$ is defined as the sum of errors across all terminal nodes:

2 $$R\left(T\right)=\sum_{i=1}^{\widetilde{T}}R\left({t}_{i}\right)$$

where $\widetilde{T}$ represents the set of terminal nodes of the tree T.

EAT selects the optimal combination $\left({x}_{j},{s}_{j}\right)$ by minimizing the sum of errors:

3 $$R\left({t}_{L}\right)+R\left({t}_{R}\right)=\frac{1}{n}\sum_{\left({{\varvec{x}}}_{{\varvec{i}}},{{\varvec{y}}}_{{\varvec{i}}}\right)\in {t}_{L}}\sum_{r=1}^{s}{\left({y}_{ri}-{y}_{r}\left({t}_{L}\right)\right)}^{2}+\frac{1}{n}\sum_{\left({{\varvec{x}}}_{{\varvec{i}}},{{\varvec{y}}}_{{\varvec{i}}}\right)\in {t}_{R}}\sum_{r=1}^{s}{\left({y}_{ri}-{y}_{r}\left({t}_{R}\right)\right)}^{2}$$

Here, n represents the sample size, ${y}_{r}\left({t}_{L}\right), {y}_{r}\left({t}_{R}\right)$ denote the estimated outputs ${y}_{r},(r=1,\dots ,s)$ for left and right child nodes (${t}_{L}$,${t}_{R}$), respectively. The threshold ${s}_{j}$ satisfies ${s}_{j}\in {S}_{j}$. 5

Within the EAT framework, the estimated output of the left child node is defined as ${y}_{r}\left({t}_{L}\right)=\text{max}\{\mathit{max}\{{y}_{ri}:\left({{\varvec{x}}}_{i}, {{\varvec{y}}}_{i}\right)\in {t}_{L}\}{, y}_{r}\left({I}_{T\left(k|{t}^{*}\to {t}_{L},{t}_{R}\right)}({t}_{L})\right)\}$, and the estimated output of the right child node is defined as ${y}_{r}\left({t}_{R}\right)=\text{max}\{\mathit{max}\{{y}_{ri}:\left({{\varvec{x}}}_{{\varvec{i}}}, {{\varvec{y}}}_{i}\right)\in {t}_{R}\}{, y}_{r}\left({I}_{T\left(k|{t}^{*}\to {t}_{L},{t}_{R}\right)}({t}_{R})\right)\}$.

Here,${y}_{r}\left({I}_{T\left(k|{t}^{*}\to {t}_{L},{t}_{R}\right)}({t}_{L})\right)=\text{max} {\{y}_{r}\left({t}{\prime}\right):{t}{\prime}\in \left({I}_{T\left(k|{t}^{*}\to {t}_{L},{t}_{R}\right)}\left({t}_{L}\right)\right), {y}_{r}\left({t}{\prime}\right)\},$ where ${y}_{r}\left({t}{\prime}\right)$ represents the estimated output variable ${y}_{r} (r=1,\cdots , s)$ at node ${t}{\prime}$. ${I}_{T\left(k|{t}^{*}\to {t}_{L},{t}_{R}\right)}\left({t}_{L}\right)$ signifies the set of terminal nodes created after the k-th Pareto optimal split from node ${t}_{L}$. 6

In the dataset $\aleph$, the set of terminal nodes determined by the EAT algorithm for tree ${T}^{EAT}\left(\aleph \right)$ is typically denoted as ${\widetilde{T}}^{EAT}\left(\aleph \right)$. Based on this definition, the estimation of the output vector corresponding to the input vector ${{\varvec{x}}}{\prime}$ can be expressed as ${\varvec{d}}_{{T^{EAT} \left( \aleph \right)}} \left( {\user2{x^{\prime}}} \right): = {\varvec{y}}\left( {t^{\prime}} \right)$ (2), where ${{\varvec{x}}}{\prime}\in supp\left({t}{\prime}\right)$ and ${t}{\prime}\in {\widetilde{T}}^{EAT}\left(\aleph \right)$.

Esteve et al. [11] suggest that deep trees can be pruned using cross-validation methods, similar to those proposed by Breiman et al. [36]. Let ${T}^{EAT*}\left(\aleph \right)$ denote the optimal tree obtained through the algorithm, and define the multidimensional estimator as ${{\varvec{d}}}_{{T}^{EAT*}\left(\aleph \right)}\left({\varvec{x}}\right)$. The technology derived can then be expressed as:

4 $${{\varvec{d}}}_{{T}^{EAT*}\left(\aleph \right)}\left(x\right)\text{ as }{\widehat{\Psi }}_{{T}^{EA{T}^{*}}\left(\aleph \right)}=\left\{\left({\varvec{x}},{\varvec{y}}\right)\in {R}_{+}^{m+s} :{\varvec{y}}\le {{\varvec{d}}}_{{T}^{EA{T}^{*}}\left(\aleph \right)}\left({\varvec{x}}\right)\right\}$$

The EAT efficiency score $\varnothing \left({{\varvec{x}}}_{k},{{\varvec{y}}}_{k}\right)$ can be derived by substituting ${\widehat{\Psi }}_{{T}^{EA{T}^{*}}\left(\aleph \right)}$ for $\Psi$ in equation $\phi \left({{\varvec{x}}}_{k},{{\varvec{y}}}_{k}\right)=\text{max}\left\{{\phi }_{k}\in R:\left({{\varvec{x}}}_{k},{\phi }_{k}{{\varvec{y}}}_{k}\right)\in \Psi \right\}$. Esteve et al. [11] have proven that $\varnothing \left({{\varvec{x}}}_{k},{{\varvec{y}}}_{k}\right)$ is determined through the optimization model described below.

5 $${\varnothing }^{EAT}\left({{\varvec{x}}}_{k},{{\varvec{y}}}_{k}\right)={max}_{s.t.}{\phi }_{k}$$

$$\text{s}.\text{t}. \sum_{t\in {\widetilde{T}}^{EA{T}^{*}}\left(\aleph \right)}{\lambda }_{t}{{\varvec{a}}}_{j}^{t}\le {x}_{{k}_{j}}, j=1\dots ,m$$

$$\sum_{t\in {\widetilde{T}}^{EA{T}^{*}}\left(\aleph \right)}{\lambda }_{t}{d}_{r{T}^{EAT*}\left(\aleph \right)}({{\varvec{a}}}^{t})\ge {\phi }_{k}{y}_{rk}, r=1\dots ,s$$

$$\sum_{t\in {\widetilde{T}}^{EA{T}^{*}}\left(\aleph \right)}{\lambda }_{t}=1, {\lambda }_{t}\in \left\{\text{0,1}\right\}, i=1,\dots ,n$$

**Fig. 1:**
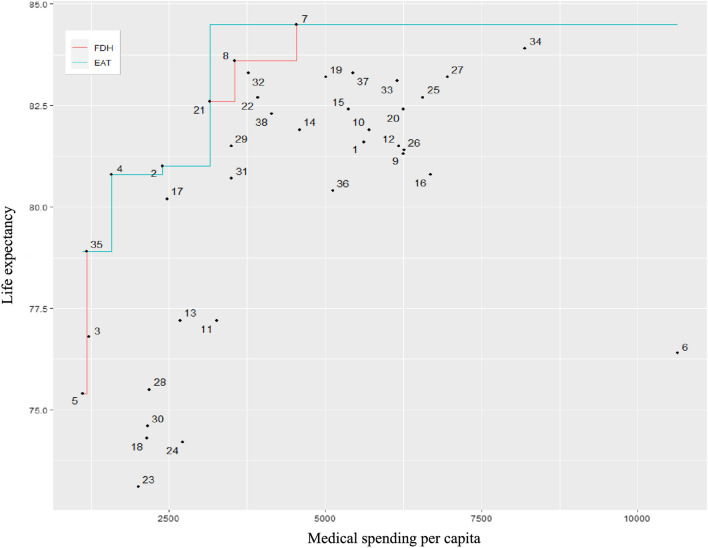
Comparison of the minimum extrapolation principle

In Fig. 1, both FDH and EAT techniques generate stepwise functions as estimates of the production frontier when there is a single input variable. However, EAT does not adhere to the minimum extrapolation principle. Furthermore, simulations have demonstrated that EAT exhibits superior discriminatory power among efficient DMUs and reduces inefficiency underestimation, providing a closer approximation to the actual production frontier compared to FDH [11].

## Random forest for efficiency analysis trees (RFEAT)

Given a training sample $\aleph$ of size n, Random Forest (RF) performs resampling to create bootstrap samples, each of the same size as ℵ. Each bootstrap sample is used to train a single decision tree. The aggregation of individual bootstrap outcomes is referred to as bagging. However, the RF algorithm does not employ simple bagging, which uses all input variables at each split. Instead, the tree training process is modified to randomly select a subset of input variables at each candidate split. This procedure is designed to reduce inter-correlation among trees. If a subset of input variables strongly predicts the output variable, these variables are likely to be selected across many trees, potentially leading to inter-correlation among the trees. By synthesizing these less correlated random models, RF reduces generalization error. Combining information from these diverse random models typically yields better predictive performance than relying on a single non-random model [39].

Esteve et al. [12] introduce the RFEAT method, which estimates the frontier using the RF algorithm. In RFEAT, the pruning process through cross-validation used in EAT is replaced by a dual randomization technique, which involves bootstrapping the data and randomly selecting subsets of input variables at each split, followed by the aggregation of predictions from all trees. By using a large number of trees, RFEAT addresses the issues of inefficiency underestimation and mitigates the instability associated with using a single tree, thereby achieving a significant reduction in variance of the efficiency estimates.

The advantage of ensemble methods like RF lies in their ability to estimate generalization error using the out-of-bag(OOB) samples $\aleph /{\aleph }_{q}$. In RF, OOB estimates for an observation $\left({{\varvec{x}}}_{i},{{\varvec{y}}}_{i}\right)$ are computed using only the model ${T}^{EAT}\left({\aleph }_{q}\right) where \left({{\varvec{x}}}_{i},{{\varvec{y}}}_{i}\right) \notin {\aleph }_{q}$. For each OOB observation, the model predicts the output value, and the OOB error is calculated as the difference between the predicted and actual value. The average of these OOB errors estimates of the generalization error.

Generalization error is useful for determining variable importance, allowing for the ranking of input variables ${x}_{i},{\cdots ,x}_{m}$. The importance of input variable ${x}_{i},{\cdots ,x}_{m}$ in RFEAT is calculated as follows: A new database ${\aleph }^{j}$ is created by randomly permuting the values of variable ${x}_{j}$, and RFEAT technique is applied on the training sample ${\aleph }^{j}$. The generalization error (${err}^{RFEAT\left({\aleph }^{j}\right)}$) in this Random Forest is calculated as Eq. (6).

6 $$er{r}^{RFEAT({\aleph }_{j})}=\frac{1}{n}\sum_{\left({x}_{i},{y}_{i}\right)\in {\aleph }_{j}}\sum_{r=1}^{s}{\left({y}_{ri}-{y}_{r}^{RFEAT\left({\aleph }^{j}\right)}({{\varvec{x}}}_{i})\right)}^{2}$$

Here, ${y}_{r}^{RFEAT\left({\aleph }^{j}\right)}({{\varvec{x}}}_{i})$ is the r-th element of vector ${{\varvec{y}}}_{r}^{RFEAT\left({\aleph }^{j}\right)}({{\varvec{x}}}_{i})$ = $\frac{1}{\left|{K}_{i}\left({\aleph }^{j}\right)\right|}\sum_{q\in {K}_{i}\left({\aleph }^{j}\right)}{d}_{{T}^{EAT}\left({\aleph }_{q}\right) }({{\varvec{x}}}_{i})$. $${K}_{i}\left({\aleph }^{j}\right)= \left\{\begin{array}{c}q:q=1,\dots ,p, \left({{\varvec{x}}}_{i},{{\varvec{y}}}_{i}\right)\notin {\aleph }_{q}\}\end{array}\right.$$ and $\left|\cdot \right|$ define the ordinality of the sets. The percentage increase in generalization error when input variable ${x}_{j}$ is randomly rearranged is calculated as follows.

7 $$\%{Inc}^{RFEAT}\left({x}_{j}\right)=100\cdot \left[\frac{{err}^{RFEAT\left({\aleph }^{j}\right)-}{err}^{RFEAT\left(\aleph \right)}}{{err}^{RFEAT\left(\aleph \right)}}\right]$$

### Hyperparameter setting

#### EAT algorithm and hyperparameter configuration

Selecting the most suitable hyperparameters is crucial as different boundaries are estimated based on these settings. The hyperparameters are as follows: First, the minimum number of observations (Numstop) required to split a node must be established. As the value of this hyperparameter increases, the size of the tree decreases. Second, to apply the cross-validation technique, it must be determined how many sets (folds) the data will be divided into. This is not directly related to the size of the tree. Third, the number of nodes between the initial node and the farthest terminal node must be limited. Introducing this parameter prevents the typical growth-pruning process, allowing the tree to grow to the necessary depth. Fourth, the maximum number of terminal nodes must be decided; the tree grows until it reaches the required number of terminal nodes and then returns, so the growth-pruning process does not occur. While deciding on the number of nodes and terminal nodes eliminates the pruning process, reducing computation time, the pruning process is preferred for inferential rather than technical objectives. In this study, optimization was performed for each hyperparameter.

The selected hyperparameters are used to create a combination grid that determines the number of models to be fit. For instance, with parameters numStop = {3, 5, 7} and fold = {5, 7}, six different models are configured and fitted: {numStop = 3, fold = 5}, {numStop = 3, fold = 7}, {numStop = 5, fold = 5}, {numStop = 5, fold = 7}, {numStop = 7, fold = 5}, and {numStop = 7, fold = 7}.

#### RFEAT algorithm and hyperparameter settings

The RFEAT algorithm operates as follows: In the first step, the number of trees ($p$) that constitute the forest is determined. Subsequently, $p$ random subsamples are generated from the original dataset with replacement using the bootstrap method. The EAT algorithm is then applied to each subsample without pruning, and a stopping rule ($n\left(t\right)\le {n}_{\text{min}}$) is employed, where ${n}_{min}$ serves as a tunable hyperparameter. During the execution of the EAT algorithm, each time a split routine is initiated, a subset of input variables is randomly selected fr

om the full set of input variables. The number of variables to be randomly selected (mtry) is also treated as an additional hyperparameter.

To determine the number of randomly selected variables (mtry), one of the following five rules is used (all values derived from the rules are floored for use, where m is the total number of input variables, s is the number of output variables, and n(t) is the sample size of the parent node):

**Table:** 

1)	Breiman’s Rule: mtry = m/3
2)	Rule DEA1: mtry = n(t)/2−s
3)	Rule DEA2: mtry = n(t)/3−s
4)	Rule DEA3: mtry = n(t)/2 s
5)	Rule DEA4: mtry = min{n(t)/s, n(t)/3−s}

Rule 1 was proposed by Breiman [16] and represents the normal value used in standard random forests for regression problems, where the number of randomly selected input variables should be one-third of the total input variables. The remaining rules stem from several empirical rules found in previous studies related to DEA, which concern the relationships between the number of DMUs and the number of inputs and outputs. Unlike Breiman’s rule, which does not depend on the parent node sample size n(t), all other rules are dependent on n(t).

[40–47]

After implementing EAT algorithm to $p$ bootstrap subsamples ${\aleph }_{1},\dots ,{\aleph }_{p}$, one must obtain $p$ fitted trees ${T}^{EAT}\left({\aleph }_{1}\right),\cdots ,{T}^{EAT}\left({\aleph }_{p}\right)$. Given input vector ${\varvec{x}}\in {R}_{+}^{m}$, the output levels corresponding to each tree are represented by ${{\varvec{d}}}_{{T}^{EAT}\left({\aleph }_{q}\right)}\left({\varvec{x}}\right)$. The final output value is determined by averaging individual estimates as follows.

8 $${\varvec{y}}^{RFEAT\left( \aleph \right)} \left( {\varvec{x}} \right): = \frac{1}{p}\mathop \sum \limits_{q = 1}^{p} {\varvec{d}}_{{T^{EAT} \left( {\aleph_{q} } \right)}} \left( {\varvec{x}} \right)$$

Using Random Forest techniques, the derived input and output sets can be defined and used as a reference set for measuring efficiency.

9 $${\widehat{\Psi }}_{RFEAT}=\left\{\begin{array}{c}\left({\varvec{x}}, {\varvec{y}}\right)\in {R}_{+}^{m+s}: y\le {{\varvec{y}}}^{RFEAT\left(\aleph \right)}(x)\}\end{array}\right.$$

${\widehat{\Psi }}_{RFEAT}$ satisfies the classical disposability in production theory [12]. The optimal combination of Random Forest hyperparameters extracted in this study is as follows: The minimum number of observations (${n}_{min}$) is 7, and the number of individual trees bootstrapped ($p$) is 500. The number of input variables selected randomly from the original set (mtry) is set at one-third of the total number of input variables (m), in accordance with Breiman’s rule.

## Data and variables

This study utilized OECD health statistics from 2017 to 2021 and the World Bank Database to measure the quality efficiency of health systems. Drawing on previous research, life expectancy in 2021 was selected as an output variable, as it is widely recognized as a representative indicator for evaluating efficiency in public health expenditure [24, 26]. Life expectancy has been consistently employed to measure the health and welfare of populations due to its broad applicability and data availability [20, 24, 26, 48].

Input variables were chosen based on prior studies and data availability. Per capita health expenditure is commonly used financial resource input for analyzing health system efficiency [25, 49]. Other frequently used variables include per capita health expenditure (both total and public) and average educational attainment [28, 50]. Research suggests that encompassing both financial resources and socio-environmental factors is essential, as population health is influenced not only by health expenditure but also by broader social determinants [26, 51].

In alignment with this approach, this study selected per capita medical spending (World Bank), public health expenditure as a share of total health expenditure (World Bank), and health spending as a percentage of GDP (WHO Global Health Expenditure) as input variables representing financial resources. To represent environmental factors, net enrollment ratio for upper secondary education (NER, UNESCO Institute for Statistics) and per capita GDP(World Bank) were included. The selection was guided by previous studies and the availability of consistent data across countries.

Certain variables, such as NER, public health expenditure share, and health spending are ratio variables. Ratio variables enable relative comparisons and are particularly useful for cross-country analyses despite differences in scale. However, recent research has criticized the use of ratio variables for introducing nonlinearity and convexity issues in the efficiency frontier of standard DEA models [52, 53]. Unlike standard DEA, the RFEAT and EAT methods employed in this study leverage decision tree-based approaches to learn complex nonlinear relationships between inputs and outputs. These methods do not require convexity assumptions and are thus free from issues associated with violations of convexity or linearity in the production possibility set. Additionally, decision tree-based methods reduce the risk of overfitting often encountered when using ratio variables.

To address outliers, short-term shocks, and missing data, average values from 2017 to 2021 were used [25]. Slovakia and the United Kingdom were excluded from the analysis due to missing higher education enrollment ratio data for the entire five-year period [54]. For Türkiye, the missing life expectancy value for 2021 was replaced with the closest available data within the analysis period.

The unit, source, and explanation of the input and output variables are summarized in Table 1. Life expectancy at birth, used as the output variable, is widely regarded as an indicator of population health and welfare. Over the past century, life expectancy has steadily increased across all countries [55], driven by improvements in clinical interventions [56–59] as well as broader structural determinants such as higher education levels, increased income, and enhanced social equality [60, 61].

**Table 1:** Definition of input/output variable & grouping variable

Variable	Unit	Role	Source	Explanation
Life expectancy	Years	Out	OECD stat	Life expectancy at birth, total
Per capita Medical spending	$	In	World Bank	Current health expenditure per capita, purchasing power parity (PPP)
Public health expenditure share	% of GDP	In	World Bank	Domestic general government health expenditure
Per capita GDP	$	In	World Bank	GDP per capita, purchasing power parity (PPP)
Net enrollment ratio (NER)	%	In	UNESCO Institute for Statistics	Total number of students enrolled for a specific level of education(Upper Secondary) within the official age group as a percentage of that population, both sexes (http://data.uis.unesco.org/index.aspx?queryid=3813#)
Health spending	% of GDP	In	WHO Global Health Expenditure	Current health expenditure database (http://apps.who.int/nha/database) Retrieved on April 7, 2023
Out-of-pocket expenditure Per capita	Current international $	gv	WHO Global Health Expenditure	Health expenditure through out-of-pocket payments per capita in international dollars at purchasing power parity(PPP), 2020
Ratio of old population	% of total Population	gv	OECD stat	Ratio of Population: 65 years old and over, 2021

$ = current international $ / in = input variable, out = output variable, gv = grouping variable

A total of 36 OECD member countries were selected as the subjects of analysis, and the descriptive statistics for each variable are as presented in Table 2.

**Table 2:** summary statistics of input and output variables

Variable	Obs	Mean	Std. dev	Min	Median	Max
Life expectancy, 2021	36	80.31	3.21	73.10	81.45	84.50
Five-year average Per capita medical spending	36	4,321	2,154	1,123	4,033	10,638
Five-year average Public health expenditure share	36	6.39	1.87	2.85	6.43	9.35
Five-year average per capita GDP	36	47,201	19,653	15,515	44,033	120,461
Five-year average NER	36	92.85	5.35	73.16	94.56	99.48
Five-year average health spending	36	9.05	2.38	4.32	9.13	17.23
Per capita out of pocket expenditure, 2020	36	735.44	302.88	181.51	747.55	1865.82
Ratio of old population, 2021	36	18.014	4.60	7.90	19.20	28.90

## Application in OECD countries

### Importance evaluation of input variables

**Fig. 2:**
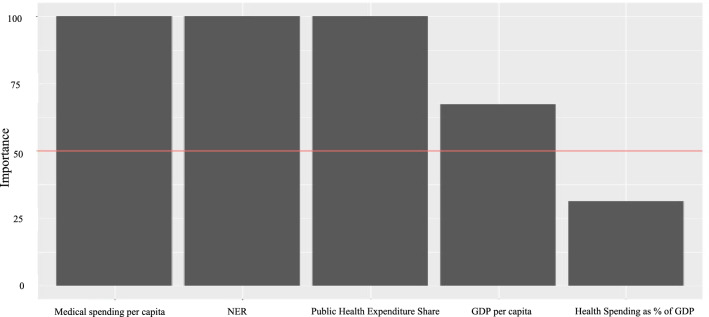
Importance of variables of EAT

EAT and RFEAT identify which input variables are most significant in research. In the EAT, variable importance is primarily determined by calculating the reduction in mean squared error (MSE) achieved by splits using a specific variable and aggregating the contributions of MSE reduction across the entire tree. The importance score of each input variable is normalized to allow comparison, with the most important variable always assigned a value of + 100, while other variables receive scores between 0 and 100. The EAT importance of the variables are illustrated in Fig. 2.

**Fig. 3:**
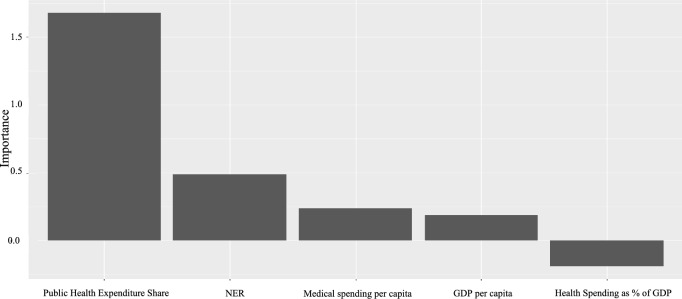
Importance of variables of RFEAT

The RFEAT also identifies the most significant input variables. The importance of input variables is evaluated based on how much the model’s generalization error increases when the input variable ${x}_{j}$ is randomly permuted [36]. In the REEAT, the input variable ${x}_{j}$ is randomly permuted in each iteration, resulting in varying importance levels for the variables. Figure 3 shows the RFEAT importance of the variables.

According to the EAT importance analysis results, Medical Spending per Capita, Net Enrollment Ratio (NER), and Public Health Expenditure Share exhibit importance values approaching 100, while Per Capita GDP slightly exceeds 62.5, and Health Spending as a Percentage of GDP slightly exceeds 25. For RFEAT, the input variables are randomly permuted during the analysis, resulting in variability in the importance values. In Fig. 3, the Public Health Expenditure Share demonstrates the highest importance among the input variables.

### EAT & RFEAT analysis results

#### EAT analysis results

**Fig. 4:**
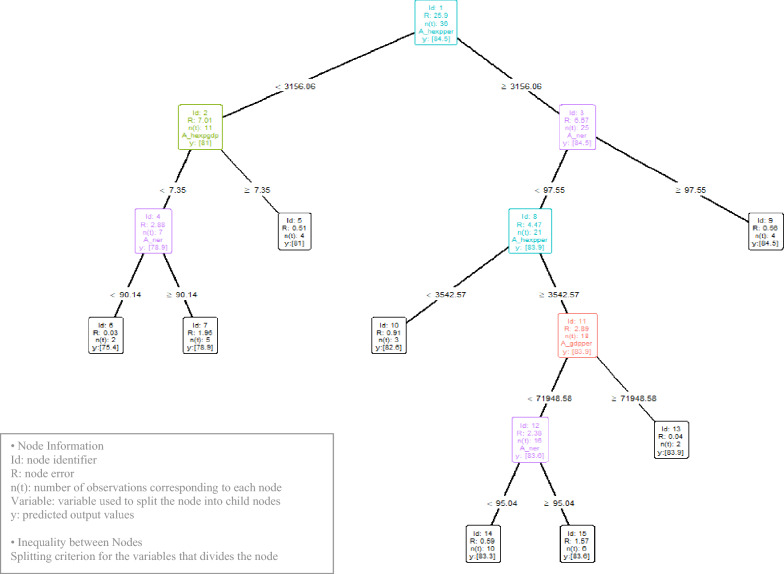
Decision tree derived from EAT

In EAT, a sensitivity analysis is initially conducted, followed by the application of the selected hyperparameter combination to produce the analysis results. Among 27 hyperparameter combinations, the one with the smallest Root Mean Squared Error (RMSE) was chosen. Figure 4 displays the decision tree results for the hyperparameter combination {Numstop, fold, max.depth} = {5, 3, 7}, which includes the minimum number of observations per node (Numstop), the number of subsets for cross-validation (fold), and the number of nodes between the root node and the furthest terminal node (max.depth).

In each node, 'id' represents the node identifier, 'R' indicates the node error, 'n(t)' denotes the number of observations corresponding to each node, 'variable' refers to the variable used to split the node into child nodes, and 'y' represents the predicted output values.

The Decision Tree in Fig. 4,^7^ comprises 7 interior nodes and 8 terminal nodes, totaling 15 nodes. Summary statistics for each node, including Node id(Node), Number of observations(n), Observation Percentage (%), Mean, Variance(Var.), and RMSE, are presented in Table 3. The error for the Decision Tree in Fig. 4 is computed as the sum of the errors of the terminal nodes, which totals 6.16.

**Table 3:** Summary statistics for nodes of the decision tree in Fig. 4

Node	n(t)	%	Mean	Var	Min	Q1	Median	Q3	Max	RMSE
1	36	100	80.47	9.9	73.1	78.48	81.5	82.7	84.5	5.09
2	11	31	77.04	7.95	73.1	74.85	76.8	79.55	81	4.79
3	25	69	81.98	3.36	76.4	81.5	82.4	83.2	84.5	3.1
4	7	19	75.51	3.89	73.1	74.25	75.4	76.35	78.9	3.85
5	4	11	79.7	3.85	76.8	79.35	80.5	80.85	81	2.14
6	2	6	74.85	0.61	74.3	74.57	74.85	75.12	75.4	0.78
7	5	14	75.78	5.38	73.1	74.2	75.5	77.2	78.9	3.75
8	21	58	81.86	3.68	76.4	81.4	82.4	83.2	83.9	2.77
9	4	11	82.58	1.78	81.5	81.8	82.15	82.93	84.5	2.24
10	3	8	80.17	7.5	77.2	78.95	80.7	81.65	82.6	3.3
11	18	50	82.14	2.86	76.4	81.53	82.55	83.2	83.9	2.4
12	16	44	82	2.99	76.4	81.47	82.35	83.2	83.6	2.32
13	2	6	83.3	0.72	82.7	83	83.3	83.6	83.9	0.85
14	10	28	82.13	0.85	80.8	81.43	81.95	83.08	83.3	1.46
15	6	17	81.78	7.34	76.4	82.03	82.75	83.25	83.6	3.07

Derived using EAT algorithm

The decision tree diagram and summary statistics facilitate understanding the splitting process of each node. For instance, the root node id = 1 encompasses all 36 observations. This node splits into child nodes 2 and 3, where the splitting variable, per capita medical expenditure at a split point of s = 3156.06, minimizes the sum of errors of the child nodes, recorded as 13.68.

The production frontier can be discerned through the terminal nodes. Among the 36 OECD countries analyzed, 4 are estimated to have a life expectancy of 81 years, while 2 are at 75.4 years. Others are estimated as follows: 5 countries at 78.9 years, 4 at 84.5 years, 3 at 82.6 years, 2 at 83.9 years, 10 at 83.3 years, and 6 at 83.6 years. Table 4 shows the corresponding countries in the form of ISO 3166–1 A-3 Country Code.

**Table 4:** Summary of results for terminal nodes of the decision tree in Fig. 4

Node ID	N(t)	%	Life expectancy	R(t)	ISO 3166-1 A-3 Country Code Corresponding to the Node
5	4	11	81	0.51	CHL COL CRI GRC
6	2	6	75.4	0.03	MEX HUN
7	5	14	78.9	1.95	EST LVA LTU POL TUR
9	4	11	84.5	0.56	JPN BEL IRL PRT
10	3	8	82.6	0.91	CZE ISR SVN
13	2	6	83.9	0.04	LUX CHE
14	10	28	83.3	0.59	CAN AUT DNK DEU ISL ITA NLD NOR ESP NZL
15	6	17	83.6	1.57	USA KOR FIN FRA SWE AUS

Derived using EAT algorithm

#### RFEAT out-of-bag (OOB) error convergence

**Fig. 5:**
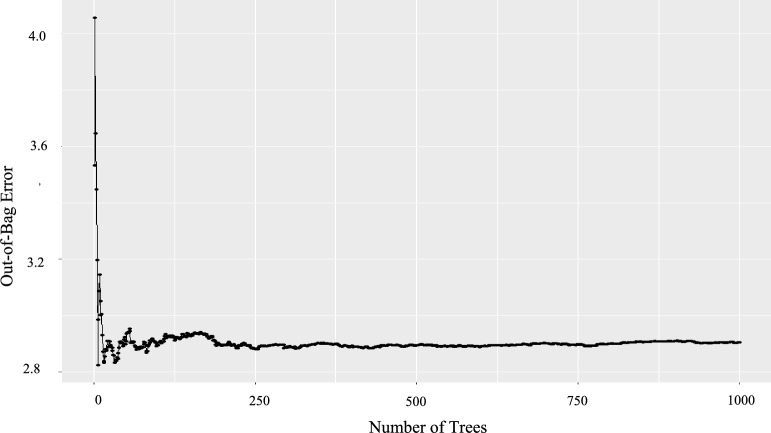
Out-of-bag (OOB) error

In the sensitivity analysis of RFEAT, the hyperparameter combination that recorded the smallest RMSE (1.90) included a minimum observation count of 5, a total of 1000 bootstrapped individual trees, and a selection of input variables randomly chosen from the original dataset in accordance with the DEA Rule 1, which mandates that the number of observations should be at least twice the number of variables selected. Figure 5 illustrates the OOB error for the ensemble consisting of 1000 trees.

For a small number of trees, the OOB error exhibits significant volatility but generally demonstrates a converging trend. As shown in Fig. 5, the OOB error converges around a level of approximately 2.9 with more than 250 trees. The overall error rate for this forest was recorded at 8.4413.

### Efficiency metrics and graphs

In this study, we estimate efficiency using an output-oriented model based on the assumption of Variable Returns to Scale (VRS). Given the uncertain nature of the scale of returns in health systems across different countries, a more flexible assumption of VRS is chosen over Constant Returns to Scale (CRS).

Assuming an output-oriented VRS model, we calculated the technical efficiency for each OECD country. It is important to note that technical efficiency measured by output-oriented methods may differ from those measured by input-oriented methods. Output-oriented efficiency scores are interpreted such that higher scores indicate inefficiency. By taking the reciprocal for convenience, it could be interpreted as inefficient if the value approaches zero, and efficient if the value approaches 1. Table 5 presents the efficiencies estimated using the output-oriented FDH, EAT, and RFEAT models under VRS assumptions, the reciprocals of these efficiencies, and the rankings derived from these reciprocals.

**Table 5:** Technical efficiency of each nation in OECD (output-oriented, VRS)

	Country	FDH			EAT			RFEAT		
		$\varnothing$	$1/\varnothing$	Rank	$\varnothing$	$1/\varnothing$	Rank	$\varnothing$	$1/\varnothing$	Rank
1	Canada	1.000	1.000	1	1.021	0.980	20	1.022	0.978	22
2	Chile	1.000	1.000	1	1.000	1.000	1	1.004	0.996	12
3	Colombia	1.000	1.000	1	1.055	0.948	32	1.035	0.967	28
4	Costa Rica	1.000	1.000	1	1.003	0.998	11	0.993	1.007	3
5	Mexico	1.000	1.000	1	1.000	1.000	1	1.025	0.976	24
6	United States	1.094	0.914	36	1.094	0.914	36	1.099	0.910	36
7	Japan	1.000	1.000	1	1.000	1.000	1	0.995	1.005	4
8	Korea, Rep	1.000	1.000	1	1.000	1.000	1	0.992	1.008	1
9	Austria	1.023	0.977	31	1.025	0.976	26	1.028	0.973	27
10	Belgium	1.021	0.980	26	1.032	0.969	29	1.025	0.976	25
11	Czechia	1.047	0.955	33	1.070	0.935	34	1.070	0.935	33
12	Denmark	1.021	0.980	28	1.022	0.978	23	1.024	0.977	23
13	Estonia	1.022	0.978	29	1.022	0.978	22	1.042	0.960	30
14	Finland	1.021	0.980	26	1.021	0.980	20	1.020	0.981	21
15	France	1.015	0.986	24	1.015	0.986	18	1.016	0.985	19
16	Germany	1.010	0.990	23	1.031	0.970	28	1.036	0.965	29
17	Greece	1.000	1.000	1	1.010	0.990	15	1.007	0.993	13
18	Hungary	1.015	0.985	25	1.015	0.985	19	1.066	0.938	32
19	Iceland	1.000	1.000	1	1.001	0.999	9	0.998	1.002	9
20	Ireland	1.000	1.000	1	1.026	0.975	27	1.010	0.990	16
21	Israel	1.000	1.000	1	1.000	1.000	1	0.997	1.003	8
22	Italy	1.000	1.000	1	1.007	0.993	14	1.008	0.993	14
23	Latvia	1.079	0.927	35	1.079	0.927	35	1.081	0.925	34
24	Lithuania	1.063	0.940	34	1.063	0.940	33	1.084	0.922	35
25	Luxembourg	1.000	1.000	1	1.015	0.986	17	0.997	1.003	7
26	Netherlands	1.023	0.977	30	1.023	0.977	24	1.026	0.975	26
27	Norway	1.000	1.000	1	1.001	0.999	9	1.004	0.996	11
28	Poland	1.045	0.957	32	1.045	0.957	31	1.051	0.952	31
29	Portugal	1.000	1.000	1	1.037	0.965	30	1.012	0.988	17
30	Slovenia	1.001	0.999	19	1.024	0.977	25	1.018	0.982	20
31	Spain	1.000	1.000	1	1.000	1.000	1	0.996	1.004	6
32	Sweden	1.006	0.994	22	1.006	0.994	13	1.010	0.990	15
33	Switzerland	1.000	1.000	1	1.000	1.000	1	0.993	1.007	2
34	Türkiye	1.000	1.000	1	1.000	1.000	1	0.995	1.005	5
35	Australia	1.004	0.996	20	1.004	0.996	12	1.004	0.997	10
36	New Zealand	1.005	0.995	21	1.012	0.988	16	1.013	0.987	18
Mean			1.014	0.986		1.022	0.979		1.022	0.979
SD			0.023	0.022		0.024	0.022		0.028	0.026

EAT efficiency was determined using the hyperparameter combination {NumStop, fold, max.depth} = {5, 3, 5}, which yielded the smallest RMSE (3.02) in the EAT sensitivity analysis. Similarly, RFEAT efficiency was calculated using the hyperparameter combination {NumStop, m, s_mtry} = {5, 1000, 1}, which recorded the smallest RMSE (1.90) in the sensitivity analysis.

In the case of the FDH model, the number of countries achieving an efficiency score of 1 totals 18, whereas for the EAT model, it is 8, and for RFEAT, only 1 country achieves this score. This pattern indicates that RFEAT, followed by EAT, and then FDH, possesses a greater ability to distinguish the efficiency of countries. Notably, RFEAT demonstrates efficiency values below 1 for some countries, a phenomenon occurring because the boundaries derived through the RFEAT method do not envelop all observations. The bagging process in RFEAT uses various subsets of randomly selected data from the original dataset to fit individual tree models. Consequently, each DMU only appears in certain sets of these random samples, and not all decision-making units are enveloped by each boundary. Therefore, efficiency values less than 1 can emerge in the final computation, suggesting that DMUs with values less than 1 are likely to be particularly efficient [12].

The reciprocal of the efficiency scores for individual countries can be interpreted similarly to input-oriented models, where a higher score indicates greater efficiency and a lower score indicates inefficiency. In RFEAT, the highest efficiency score is for South Korea (1.008), followed by Switzerland (1.007), and Costa Rica (1.007). The lowest efficiency score is observed for the United States (0.910), with Lithuania being the second lowest (0.922). According to EAT results, the highest efficiency scores are for Chile, Mexico, Japan, South Korea, Israel, Spain, Switzerland, and Türkiye. The lowest scores are again for the United States (0.914) and Latvia (0.927).

**Fig. 6:**
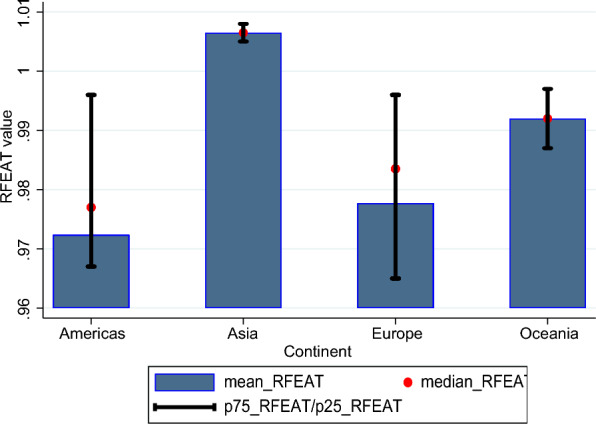
Comparison by groups based on the continents

**Fig. 7:**
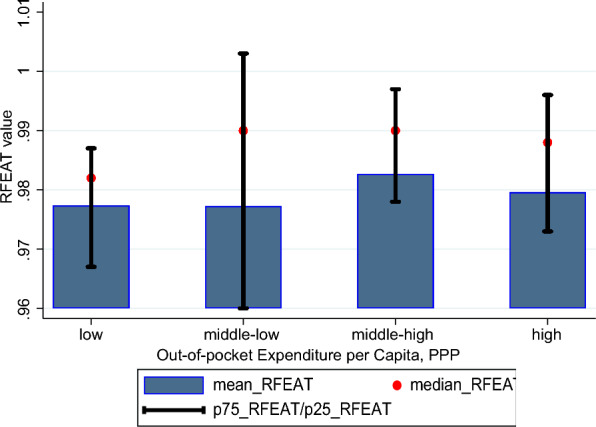
Comparison by groups based out-of-pocket expenditure per capita, PPP

**Fig. 8:**
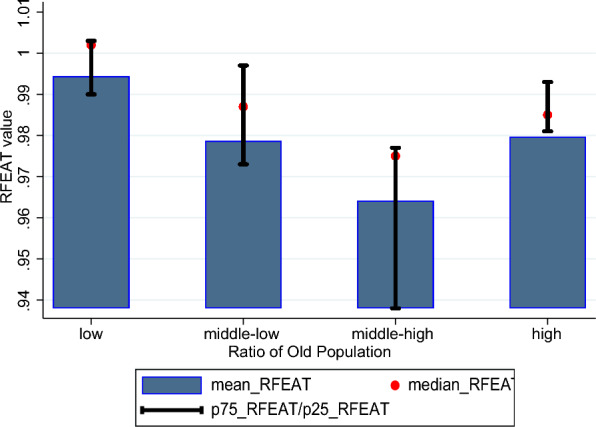
Comparison by groups based old population ratio (65 and over)

The analysis of the inverse of the derived RFEAT efficiency scores ($1/\varnothing$) across different groups is illustrated in Figs. 6, 7, and 8. Figure 6 compares the RFEAT efficiency scores by continent, while Fig. 7 presents a comparison of RFEAT efficiency scores across four groups based on levels of out-of-pocket expenditure per capita. Figure 8 compares RFEAT efficiency scores across four groups categorized by the proportion of elderly population.

The results depicted in the graphs in Figs. 6, 7, and 8 show that, on average, the health system efficiency is highest in Asian countries, followed by Oceania, Europe, and the Americas. While the two groups with higher out-of-pocket expenditures demonstrate slightly higher average efficiency compared to the two groups with lower expenditures, the differences in efficiency across these groups are minimal. Lastly, when examining the results based on elderly population ratios, the group with the lowest proportion of elderly individuals in the total population exhibits the highest average health system efficiency. The group with the second-highest proportion of elderly individuals shows the lowest average health system efficiency.

The following Table 6 presents the correlation coefficients of efficiency scores and the correlation coefficients of efficiency rankings across FDH, EAT, and RFEAT.

**Table 6:** Correlation coefficient of efficiency scores and rankings of FDH & EAT & RFEAT

	FDH & EAT	FDH & RFEAT	EAT & RFEAT
Correlation coefficient of efficiency score	0.8561	0.8840	0.8813
Correlation coefficient of efficiency ranking	0.6852	0.7842	0.8286

Calculated by Author

The correlation coefficient for efficiency scores between FDH and EAT is 0.8561, between FDH and RFEAT is 0.884, and between RFEAT and EAT is 0.8813. For rankings, the correlation coefficient between FDH and EAT is 0.6852, between FDH and RFEAT is 0.7842, and between RFEAT and EAT is 0.8286 These correlations suggest that there is over 80% similarity in efficiency scores among FDH, EAT, and RFEAT, showing minor differences. However, while the efficiency rankings between RFEAT and EAT are over 80% similar, those between FDH and EAT, as well as FDH and RFEAT, are below 80%, indicating potential inaccuracies in efficiency rankings derived using the FDH method which has inefficiency underestimation problem.

**Fig. 9:**
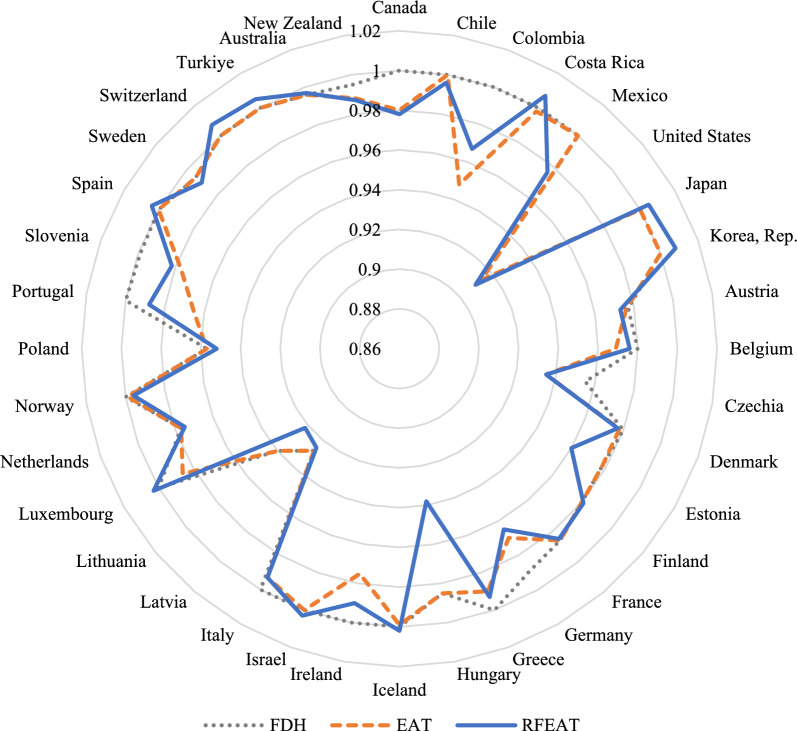
Technical efficiency of three models (output-oriented, VRS)

The results of RFEAT, EAT, and FDH estimated using the output-oriented VRS model can be represented in a radial graph as shown in Fig. 9.

RFEAT and EAT have shown greater discriminatory power compared to FDH. For instance, in the segment from Canada to Costa Rica, while the FDH model assigns values close to 1 for almost all DMUs, RFEAT and EAT produce values less than 1, indicating a greater ability to differentiate between efficient DMUs. Similarly, from DMU Denmark to Greece, RFEAT and EAT also display larger differences in DMU efficiencies compared to FDH. These results suggest that EAT and RFEAT are mitigating inefficiency underestimation inherent in FDH.

**Fig. 10:**
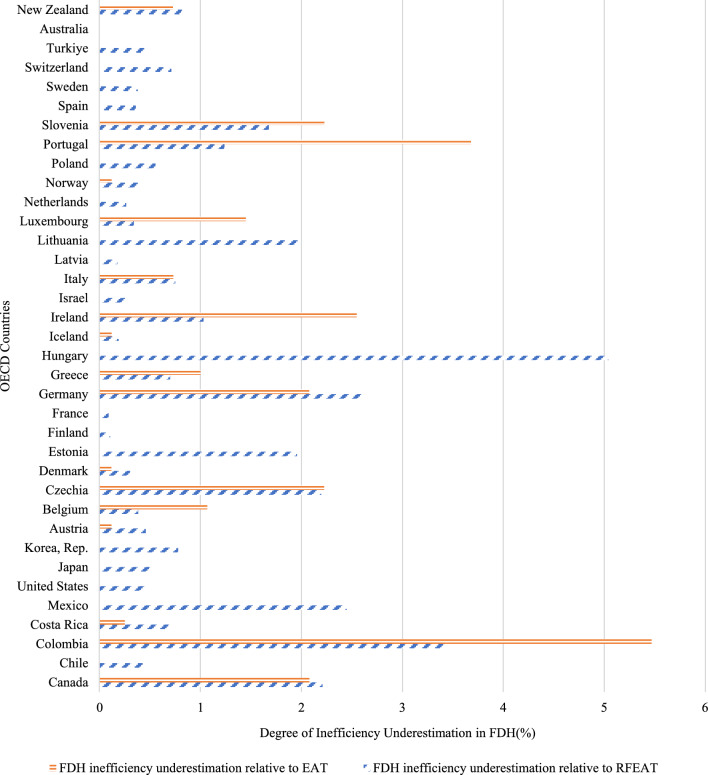
Degree of inefficiency underestimation in FDH(%)

Figure 10, located after Eq. (11), illustrates the percentage of inefficiency underestimation generated by FDH compared to EAT and RFEAT when assuming output-oriented VRS model. The degree of inefficiency underestimation in FDH based on EAT can be calculated as Eq. (10).

10 $${\text{Degree of inefficiency underestimation in FDH relative to EAT }} = \frac{{\left( {\frac{1}{{efficiency\,by\,FDH}}} \right) - \left( {\frac{1}{{efficiency\,by\,EAT}}} \right)}}{{\left( {\frac{1}{{efficiency\,by\,EAT}}} \right)}} \times 100$$

The degree of inefficiency underestimation in FDH based on RFEAT can be computed as Eq. (11).

11 $${\text{Degree of inefficiency underestimation in FDH relative to RFEAT }} = \frac{{\left( {\frac{1}{{efficiency\,by\,FDH}}} \right) - \left( {\frac{1}{{efficiency\,by\,RFEAT}}} \right)}}{{\left( {\frac{1}{{efficiency\,by\,RFEAT}}} \right)}} \times 100$$

According to the RFEAT criteria, Hungary exhibits the highest degree of inefficiency underestimation at 5.045%. The second highest is Colombia, where FDH has overfitted by 3.450% compared to RFEAT. The countries where FDH values and RF values are most similar are Australia (0.010%) and France (0.089%), indicating minimal difference between these methods. Under the EAT criteria, the highest inefficiency underestimation is again seen in Colombia at 5.470%, with Portugal following where FDH has overestimated by 3.680% compared to EAT. Many countries show no inefficiency underestimation according to EAT standards. Countries such as Chile, Mexico, the United States, Japan, Korea, Estonia, Finland, France, Hungary, Israel, Latvia, Lithuania, the Netherlands, Poland, Spain, Sweden, Switzerland, Turkey, and Australia all demonstrate 0% inefficiency underestimation.

**Fig. 11:**
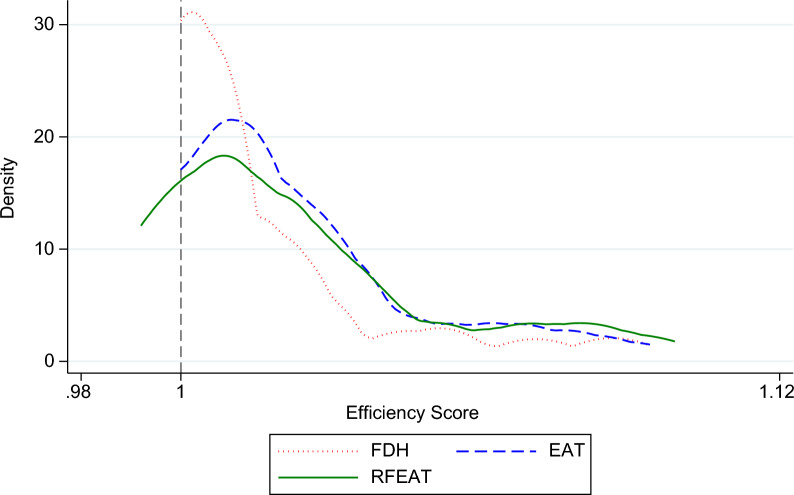
Comparison of density functions of FDH, EAT, and RFEAT

The following Fig. 11 compares the density distributions of efficiency scores obtained using FDH, EAT, and RFEAT. FDH exhibits the sharpest peak at an efficiency score of 1, indicating a higher concentration of DMUs being evaluated as fully efficient. In contrast, EAT and RFEAT demonstrate a more dispersed distribution of efficiency scores, with lower densities at 1. This dispersion suggests that EAT and RFEAT provide greater discrimination among DMUs by reducing inefficiency underestimation compared to FDH.

This result is consistent with the ability of EAT and RFEAT to address the limitations of FDH, such as the overestimation of efficiency due to its reliance on the minimal extrapolation principle. Moreover, the flatter density of RFEAT compared to EAT indicates that RFEAT may further mitigate inefficiency underestimation, likely due to the randomness and ensemble nature of its framework.

## Discussion

Previous studies have frequently employed life expectancy at birth—defined as the expected average number of years a newborn is projected to live, based on current mortality rates across all age groups—as one of the output variables for measuring the efficiency of national health systems [20, 24, 26, 48] This study similarly selects life expectancy at birth as an output variable. However, whether life expectancy at birth accurately reflects health system performance requires further scrutiny.

From a theoretical perspective, more ideal output measures could be those that reflect the quality of life, such as pain alleviation, the number of additional quality-adjusted life years (QALYs), and improvements in well-being resulting from treatment within the health system [62]. However, since these theoretically appropriate variables are not readily available in practice, output variables that can be utilized are those obtainable from organizations such as the OECD or WHO, including life expectancy, infant mortality (or survival) rates, Universal Health Coverage (UHC) and Health-Adjusted Life Expectancy (HALE). Among these, life expectancy and infant survival rates are variables with fewer missing data and are frequently updated, making them easier to incorporate into analyses. In previous studies analyzing national health systems, these two variables, either together or individually, have been the most commonly employed [20, 24, 26, 48, 63].

When comparing life expectancy at birth with infant mortality (or infant survival rates), life expectancy offers a broader perspective on the overall health of the population, as it accounts for mortality across all age groups [62]. However, its strong correlation with socio-cultural and environmental factors may distort its ability to exclusively reflect health system performance. In contrast, infant mortality (or infant survival rates) is more directly related to health system performance, particularly in terms of prenatal and neonatal care, and is closely tied to healthcare quality and hygiene standards. Nonetheless, its narrow focus on infant health limits its comprehensiveness as a general measure of health system efficiency. Furthermore, high infant mortality rates can stem from external factors, such as socioeconomic conditions, including poverty, which may not directly indicate inefficiencies within the health system [62].

Based on previous research and considering the strengths and weaknesses of each variable, this paper selects life expectancy at birth as the output variable. Despite its limitations, life expectancy effectively reflects the performance of the health system across all age groups and is available for the most recent years. In future research, to achieve a more precise estimation of efficiency, the development and collection of a broader set of output variables—capturing diverse aspects of health system outcomes—will be essential.

There has also been an ongoing debate regarding the appropriate selection of input variables in national health system efficiency analyses, as the choice and combination of input variables can significantly impact the efficiency estimation results. According to a systematic review of studies analyzing national health system efficiency, previous research has primarily employed components of the health system, health risk factors, and social determinants of health as input variables. The justification for selecting specific inputs and outputs often derives from their application in similar analyses in other contexts and the availability of relevant data [62].

Several studies advocate for incorporating both financial resources and social environmental factors, as population health is influenced not only by national health expenditure but also by broader social conditions [26, 51]. Per capita health expenditure is frequently employed as an input variable to represent financial resources in health system efficiency analyses [25, 28, 49, 50]. Furthermore, there is ongoing debate regarding whether input variables should be limited to those directly controlled by health authorities. Some studies have adopted a two-stage approach: first, estimating efficiency based on direct health inputs, and second, examining the impact of environmental variables on efficiency [24, 25].

In this study, input variables were selected based on prior research and availability including both direct factors influencing health outcomes and environmental factors, such as education levels. Moreover, the importance of the selected variables was assessed using the methods provided by EAT and RFEAT. Although there is no clear consensus in existing research on the justification for input variable selection, future studies could benefit from developing a method to identify appropriate input and output variables by incorporating the insights of health system decision-makers and practitioners [62]. This would involve determining the significance of input variables based on their relationship to output variables, and the establishment of clear criteria for the selection of variables used in both primary and secondary analyses.

Third, this study employs the average of five years' worth of data to construct input variables, with the output variable being measured from the final year. The rationale for averaging five years of data is to reflect the understanding that outcomes such as life expectancy are not simply the result of inputs from a single year, but rather an accumulation of inputs over a period of approximately five years. This approach also helps mitigate the impact of outliers or values deviating from trends due to short-term shocks and addresses years with missing data [25]. Most input variables used in this study do not have missing values for the period from 2017 to 2020, but a significant number of missing values appear in 2021, with the exception of the net enrollment ratio (NER), which only has a missing value for Japan in 2021. Similarly, in an IMF working paper, the average of five years (2013–2017) was used for input variables, with the final year’s data being applied as the output variable [25].

Finally, there may be concerns regarding the heterogeneity of DMUs in nonparametric methods. Previous studies have acknowledged this issue and, when analyzing a large number of countries, have employed clustering based on criteria such as income, using common resources within each group as inputs to ensure a minimum level of homogeneity [26, 64]. This study focuses on 36 OECD countries, but since the sample size is relatively small, further clustering based on additional criteria would reduce the number of samples. However, OECD countries are generally high- or middle-income nations, and their environments are more similar compared to non-OECD countries. Indeed, previous studies have often used clustering criteria like OECD and non-OECD when applying nonparametric methods to a large number of countries [65, 66]. While this paper does not cluster the 36 samples further, due to the small sample size, some previous studies have proposed grouping OECD and non-OECD countries based on GDP or continent for more detailed analyses [66]. 

Therefore, this study assumed the countries of OECD are approximately homogenous compared to other countries. Instead, after the efficiency scores are estimated, differences in average efficiency by group are examined. Future research could benefit from incorporating models that account for heterogeneity among countries or conducting case studies that consider the distinct institutional and environmental factors of individual countries. Such approaches may lead to more accurate analyses and the identification of appropriate strategies to enhance efficiency.

## Conclusion

This study emphasizes the importance of improving the efficiency of national health systems, especially given the increasing health expenditures across countries despite budget constraints. Enhancing health system efficiency requires the proper allocation of limited resources and improvements in health outcomes, which in turn demands accurate efficiency estimation and continuous monitoring.

In this paper, we estimated and compared the health system efficiency of 36 OECD countries using both the traditional FDH method and two machine learning techniques introduced by Esteve et al. [11, 12]. Among various machine learning methods, random forest based model was specifically chosen due to its suitability for small-to-medium sample analyses, its ability to address inefficiency underestimation, robustness against outliers and noise, and its relatively interpretable nature, including the capacity to estimate variable importance. Our findings revealed that for national health system efficiency analysis, traditional nonparametric methods like FDH tend to overestimate efficiency, particularly in settings with small sample sizes and multiple input variables. Efficiency rankings derived from the EAT and RFEAT methods showed less than 80% correlation with FDH results, with RFEAT outperforming both EAT and FDH in terms of discriminatory power and accuracy.

Based on the RFEAT method, which was identified as the most robust approach, South Korea achieved the highest efficiency score, followed by Switzerland and Costa Rica. In contrast, the USA recorded the lowest efficiency score, with Lithuania ranking second to last. Furthermore, efficiency comparisons across various groups revealed that Asian countries demonstrated the highest average health system efficiency, followed by Oceania, Europe, and the Americas. Although countries with higher out-of-pocket healthcare expenditures exhibited slightly higher efficiency, the differences across groups were minimal. Regarding the proportion of elderly individuals in the population, the group with the smallest elderly population proportion exhibits the highest average health system efficiency, whereas the group with the second-largest elderly population shows the lowest efficiency on average.

Despite the limitation of having a restricted set of available variables, this study is significant in that it introduces advanced machine learning methods to the healthcare sector, mitigating inefficiency underestimation while estimating the efficiency of OECD countries. Additionally, by utilizing clustering based on specific criteria, the analysis revealed how average efficiency differs across groups, highlighted key issues in national health system efficiency analysis and suggested directions for future research in this area. The precise efficiency analysis conducted in this study, combined with country-specific evaluations through future case studies, could provide valuable insights into the policies required by each country to improve efficiency.

Looking forward, as more health system data becomes available, the methods applied in this study may offer even more precise efficiency estimates, which can guide policymakers in improving the efficiency of national health systems. Future research could also apply these machine learning techniques to analyze resource allocation efficiency at regional levels, providing valuable insights for local health policy interventions.

## Data Availability

No datasets were generated or analysed during the current study.

## Abbreviations

ANN: Artificial neural networks
CART: Classification and regression tree
CRS: Constant returns to scale
DEA: Data envelopment analysis
DGP: Data generating process
DMU: Decision Making Unit
EAT: Efficiency analysis trees
GDP: Gross domestic product
IMF: International monetary fund
MSE: Mean squared errors
OECD: Organization for Economic Co-operation and Development
OOB: Out-of-bag
RFEAT: Random forest for efficiency analysis trees
RMSE: Root mean squared errors
SVM: Support vector machines
VRS: Variable returns to scale
WHO: World Health Organization

## Footnotes

1 The curse of dimensionality describes the phenomenon in which the data required to achieve reliable results grows exponentially as more dimensions are added.

2 Inefficiency underestimation (overfitting in DEA/FDH) arises from the structural dependency of DEA/FDH on observational data and differs fundamentally from overfitting in machine learning. While overfitting in machine learning refers to a model excessively capturing noise or intricate patterns in training data, thereby reducing generalization performance, inefficiency underestimation occurs when the minimal extrapolation principle constructs a frontier below the theoretical frontier, leading to upward bias in efficiency scores. This issue is particularly pronounced in small samples or high-dimensional settings. Mitigating overfitting can help ensure that the efficiency frontier does not overly conform to the observed data, thereby potentially reducing upward bias. However, mitigating overfitting alone is insufficient to resolve upward bias entirely; additional measures such as increasing the sample size, reducing dimensionality, or addressing noise in the data are necessary. Esteve et al. [11, 12] referred to inefficiency underestimation as overfitting, but to distinguish it from the machine learning context, this paper introduces a newly defined term.

3 The computing time of variable importance in EAT took 4 s, while it took 11 min and 24 s for RFEAT. The EAT sensitivity test took 7 s, and for RFEAT, it took 20 min and 8 s. The PC used for this analysis had the following specifications: Intel(R) Core(TM) i5-10400 CPU, 16.0 GB RAM, and a 64-bit operating system. The publicly available package EAT (https://doi.org/10.32614/RJ-2022-054) was used.

4 Recent advancements in Explainable Artificial Intelligence (XAI) techniques, such as SHapley Additive exPlanations (SHAP) and Local Interpretable Model-agnostic Explanations (LIME), have partially addressed this limitation for Neural Networks.

5 ${S}_{j}$ representing the set of potential thresholds for variable j.

6 For “x Pareto dominate y”, two conditions must be satisfied: (1) No one can strongly prefer y over x. That is, for all individuals i, ${U}_{i}\left(x\right)\ge {U}_{i}\left(y\right)$. (2) At least one individual must strongly prefer x over y. That is, for least one individual i, ${U}_{i}\left(x\right)>{U}_{i}\left(y\right)$.

7 A_NER refers to the Five-year Average Total Net Enrollment Rate for higher education, A_hexpper refers to the Five-year Average Per Capita Medical Spending, A_hexpgdp refers to the Five-year Average Health Spending as a Percentage of GDP, and A_gdpper refers to the Five-year Average Per Capita GDP.
